# Congenital Tarsal Kink Syndrome: Literature Review and Case Report

**DOI:** 10.3390/children9010031

**Published:** 2022-01-01

**Authors:** Speranța Schmitzer, Sorin-Dorin Haidu, Ioana Claudia Popteanu, Anca Angela Simionescu

**Affiliations:** 1 Department of Obstetrics and Gynecology, Carol Davila University of Medicine and Pharmacy, 050474 Bucharest, Romania; anca.simionescu@umfcd.ro; 2 Department of Ophthalmology, Emergency Clinical Eye Hospital, 010464 Bucharest, Romania; haidusorin@gmail.com (S.-D.H.); oana.popteanu@gmail.com (I.C.P.); 3 Department of Obstetrics and Gynecology, Filantropia Clinical Hospital, 011132 Bucharest, Romania

**Keywords:** congenital entropion, tarsal kink, corneal ulceration, corneal macula, ciliary edge

## Abstract

Background: The congenital tarsal kink syndrome is a rare form of congenital upper eyelid entropion associated with cardiovascular, musculoskeletal or central nervous system disorders. This syndrome must be recognized and surgically treated as a perinatal emergency to avoid associated complications—corneal ulcer, corneal leucoma, secondary amblyopia and decreased vision among children. Methods: A literature review was conducted to clarify the diagnosis particularities and the corrective surgery options of the congenital entropion on the upper eyelid. Results: Four relevant studies were found by researching the Web of Science and PubMed databases up to November 2021 for “congenital tarsal kink syndrome” and “congenital upper eyelid entropion”. Conclusions: In this paper, we present a case of congenital unilateral entropion of the upper left eyelid in the context of a tarsal kink syndrome in a one-month old infant, manifested by the absence of eyelashes on the upper eyelid of the left eye, hyperlacrimation and conjunctival hyperemia. Essential in managing the upper eyelid entropion is protecting the cornea. Furthermore, correcting a tarsal kink is eminently surgical, choosing between open or closed procedures. Herein, we address the difficulty in the timely diagnosis of this uncommon condition and make formal recommendations based on all reported cases.

## 1. Introduction

The congenital tarsal kink syndrome is a very rare form of congenital upper eyelid entropion [1,2]. Physical examination revealed the presence of a horizontal tarsal kink determining the internal rotation of the upper eyelid margin, the cilia coming into contact with the ocular globe and causing secondary blepharospasm, hyperlacrimation, photophobia and corneal ulcerations [2,3]. The average age at the time of diagnosis varies between 1 and 60 weeks [2]. It mainly affects boys and is mainly unilateral, but it can also be bilateral [2,3]. It can be associated with systemic cardiovascular conditions or central nervous system disorders [3,4,5]. The condition must be recognized and surgically treated as an emergency to avoid associated complications—corneal ulcer, corneal leucoma and secondary amblyopia [6,7,8].

This paper aims to highlight diagnostic and treatment approaches for the uncommon congenital tarsal kink syndrome and to offer arguments for universal ophthalmologic evaluation of newborns at birth.

## 2. Materials and Methods

Systematic research of the literature between 1984 and 2021 was conducted in the PubMed, Web of Science, Embase and MEDLINE (Medical Literature Analysis and Retrieval System Online) databases to select full-length articles published in peer-reviewed journals up to November 2021. The keywords included in the search strategy were “congenital tarsal kink syndrome” and “congenital upper eyelid entropion”. We limited inclusion to clinically reported cases or series of cases that fulfill the diagnosis of congenital eyelid entropion related to congenital tarsal kink syndrome, isolated or associated with a genetic or chromosomal abnormality. Full-text articles were included. We excluded articles related to other causes of congenital or noncongenital entropion, such as disinsertion of the eyelid retractors, insufficiency of verticality of the posterior lamella, spastic, involutional or cicatricial entropion.

The study was conducted in accordance with the Declaration of Helsinki, and the mother signed the informed consent for the publication of this case.

## 3. Results

We found a total of 20 relevant articles. We selected only nine full-text articles. We excluded 11 articles because no full text was available. Twenty-seven cases of congenital entropion have been identified in the context of tarsal kink syndrome, 16 male and 11 female (Table 1). The mean age of diagnosis ranges between the first week of life and 60 months. Of these patients, 77.78% (*n* = 21) of cases are unilateral and 22.23% (*n* = 6) are bilateral. The referral diagnosis was congenital entropion for thirteen cases, corneal ulceration for six cases, corneal opacity for five cases, corneal erosion for two patients and conjunctivitis for one patient. Surgical techniques included closed procedures for nine patients and open procedures for sixteen patients. An ocular patch was used in two cases.

## 4. Case Presentation

We present the case of a male two-month old patient admitted for corneal ulceration on their left eye. The pregnancy and childbirth were uneventful, the child was born at term by the vaginal way, and no associated maternal systemic diseases were reported. The mother noticed the static eyelid anomaly. The neonatologist did not consider the condition an emergency and assured the parents it would spontaneously remit. At the age of 1 month, the mother brought the child to the ophthalmologist with the following symptoms: absence of eyelashes on the upper eyelid of the left eye, hyperlacrimation and conjunctival congestion. After an examination conducted under general anesthesia, the diagnosis was corneal erosion and spastic entropion in the left eye. The recommended treatment included antibiotic and cycloplegic eye drops, a therapeutic contact lens was applied, and the patient was scheduled for a check-up after 14 days. However, as the mother noticed the spontaneous expulsion of the therapeutic contact lens a few hours after being released, the recurrence and worsening of photophobia and hyperlacrimation, associated with changes in the transparency of the cornea, the patient returned to the clinic after seven days. As a result, the decision was made to have the patient examined by the oculoplasty department.

Physical examination revealed spastic entropion of the upper eyelid on the entire eyelid length, accentuated in the inner two-third, hypertrophy of the pretarsal orbicularis muscle and the absence of the upper eyelid fold in the left eye. No static or dynamic eyelid anomalies were revealed in the right eye or in the lower left eyelid.

**Figure 1:**
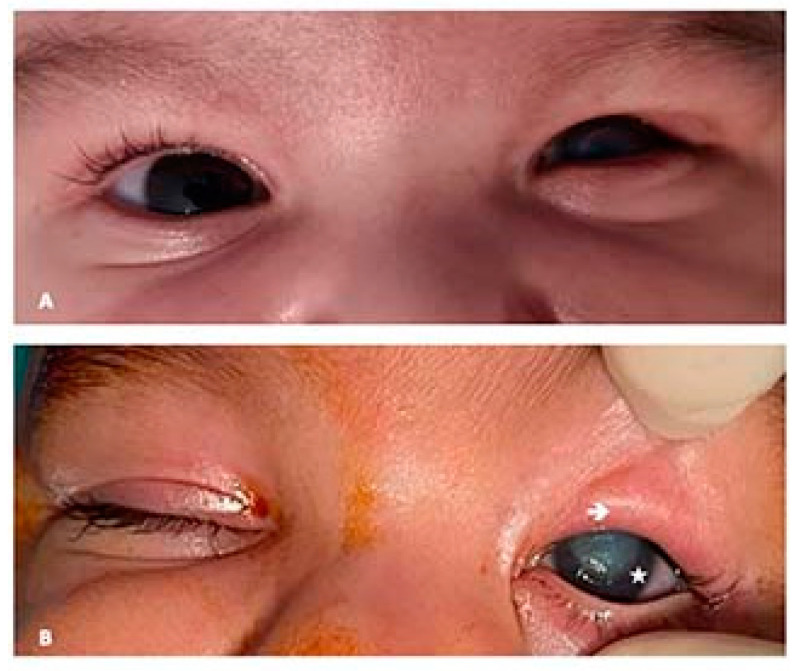
(**A**) Clinical aspect upon presentation. (**B**) Pre-op clinical aspect. To notice the absence of the cilia on the ciliary margin, the hypertrophy of the upper pretarsal orbicularis muscle and the central corneal leucoma (personal collection).

Examination under general anesthesia allowed for a difficult eversion of the upper eyelid, using the Desmarres retractor, which revealed a horizontal tarsal scar band located at approximately 2–3 mm from the edge of the eyelid, in the middle of the tarsus, accompanied by a shortening of the posterior upper eyelid lamella and the inward twisting of the ciliary margin. It also revealed a central corneal leucoma measuring approximately 6 mm in diameter and a central corneal ulceration measuring approximately 2 mm in diameter (Figure 1A,B). Applying pressure with the finger on the upper eyelid caused the eversion of the eyelid, but only for a short period, approximately 30 s, after which the entropion set back.

Upon clinical examination, the diagnosis was set—tarsal kink syndrome—and the recommendation was surgical correction. The preanesthetic pediatric visit, however, revealed an upper airways infection, so the surgery was postponed until the infection had been cleared. In the meantime, the patient underwent thorough investigations in order to exclude associated systemic pathologies, including a conjunctival smear test that later showed no pathological germs. Moreover, topical treatment was carried out at home during this time, consisting of frequent administration of corneal trophic and epithelial regeneration substances.

**Figure 2:**
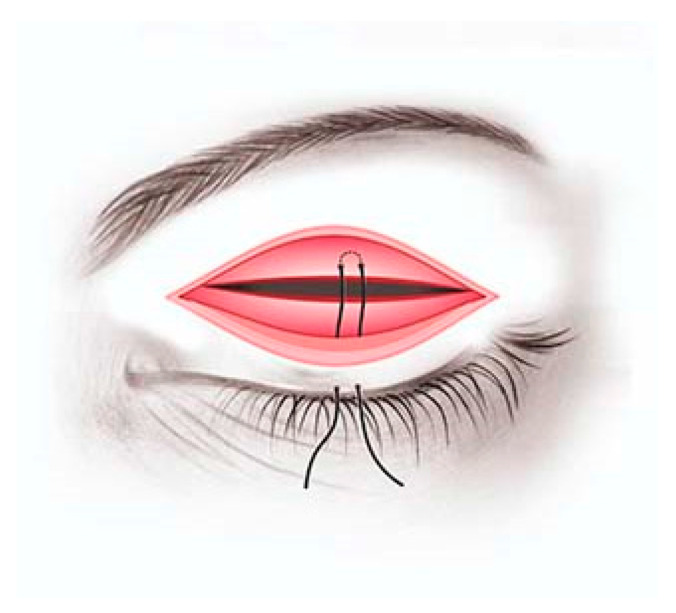
Schematic representation of the suture trajectory: skin (ciliary margin)—upper tarsal plate—skin (ciliary margin) (picture by Speranța Schmitzer).

After 14 days, the patient returned to the clinic and underwent corrective surgery of the congenital entropion on the upper eyelid, which consisted of making a cutaneous-muscular incision 2–3 mm from the ciliary margin, revealing the tarsal plate and excising the fibrotic band on the entire length and width of the tarsal plate. Since there was no resulting relaxation of the eyelid, a partial disinsertion of the levator aponeurosis was performed, at which time the eyelid returned to a normal position. Three absorbable 5/0 sutures were made on the trajectory: skin (ciliary margin)—upper tarsal plate—skin (ciliary margin) in the inner, median and outer third of the upper eyelid (Figure 2).

**Figure 3:**
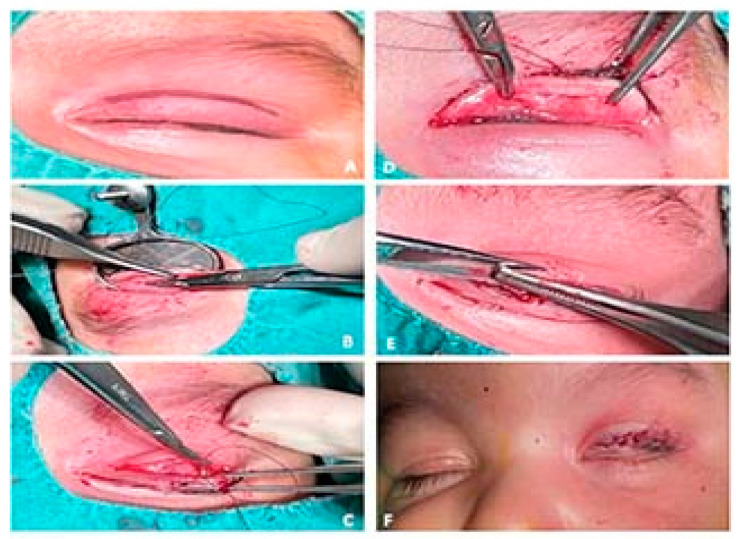
Main surgery steps. (**A**) Marking the incision 5 mm above the free eyelid edge; (**B**) The excision of the fibrous band on the entire length and width of the tarsal plate; (**C**,**D**) Application of sutures on the trajectory: skin (ciliary margin)—upper tarsal plate—skin (ciliary margin) in the inner, median and outer third of the upper eyelid; (**E**) Excision of a hypertrophied orbicular pretarsal muscle extension; (**F**) Cutaneous suture (personal collection).

The intervention was completed with the excision of a hypertrophied orbicular pretarsal muscle extension, a therapeutic contact lens placement and the execution of a blepharorrhaphy (Figure 3A–F).

The post-op evolution was favorable. Four days after surgery, the blepharorrhaphy sutures were removed and the upper eyelid retained its normal position with a slight eyelid ptosis. The recommendation was to apply a light topical anti-inflammatory steroid treatment, corneal trophic and epithelial regeneration substances with emulsified vitamin A and artificial tears with hyaluronic acid. At the one-week check-up, the upper eyelid maintained its normal position, the corneal ulceration was healed and the corneal leucoma was in remission.

At the one-month check-up, the evolution was spectacular; the eyelid maintained its normal position, the eyelid slit was symmetric to the one of the congener eye, and the cornea regained its transparency almost completely, with a persisting central corneal macula. The therapeutic contact lens was removed, and the recommendation was made to continue the topical treatment with emulsified vitamin A, artificial tears with hyaluronic acid and light anti-inflammatory steroid treatment to recover corneal transparency.

**Figure 4:**
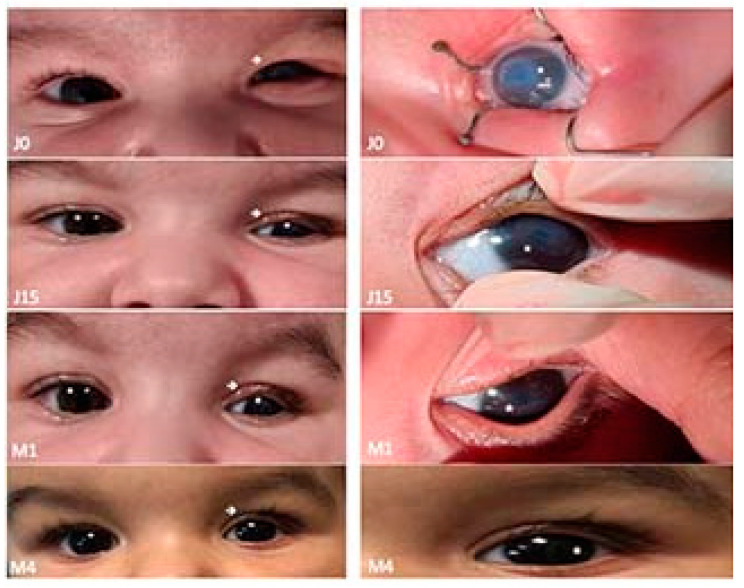
Favorable evolution and improvement in the position of the upper eyelid of the left eye and disappearance of the central corneal leucoma four months after surgery (personal collection).

One month and a half after surgery, the evolution continues to be very good, with no subjective symptomatology (Figure 4).

## 5. Discussion

Our surgical technique for congenital tarsal kink syndrome is innovative because the sutures do not include the lower edge of the tarsal plate, only the ciliary skin edge and the upper tarsal edge were associated with the excision of a hypertrophied orbicular pretarsal muscle extension.

The congenital upper eyelid entropion is rare, and, in most cases, it is associated with a shortening of the posterior eyelid lamella of different etiologies [1,2]. Although it was described as early as 1948 by Kettesy, its etiology still remains uncertain [1,3,8]. Multiple etiopathogenesis mechanisms were put forth, of which we mention the overreaction of the marginal fibers of the orbicularis muscle, causing the infolding of the tarsal plate in utero. Moreover, primary tarsal defects, disinsertion of the levator aponeurosis, eyelid disjunctions in utero and exogenous mechanical causes in utero [1,13]. Sires et al. have suggested an acute eyelid trauma during childbirth due to right occiput anterior presentation [8]. Although most cases do not associate systemic pathologies, they can appear, which is why these patients must be carefully examined by a pediatrician who would insist on the cardiovascular, musculoskeletal and nervous systems [3,4,7]. In the United Kingdom, screening for eye pathologies 72 h after birth is offered by ophthalmologists to newborns, using clinical eye examination and direct ophthalmoscopy to check for light reflex and red reflex [14]. Costs for universal newborn eye screening must be balanced against the potential cost for children’s care to improve visual outcomes in case of missed diagnosis [15].

The surgical goal in patients with tarsal kink syndrome is to obtain a normal eyelid statics. Regardless of the surgical technique used—closed or open procedures for entropion correction—the results reported in the reviewed articles are good in terms of eyelid statics. The patient’s visual prognosis is related to the timeline of diagnosis and treatment initiation, as well as the associated corneal changes (erosion, ulceration, perforation).

Most patients with tarsal kink syndrome are isolated cases that do not have another associated pathology. There are the situations when this condition can be found in some syndromes or other pathologies.

In comparison to our surgical technique, some of the techniques described in Table 1 use closed procedures with everting sutures [1,4,9], while the open procedure is also different, using a transconjunctival approach with horizontal tarsotomy and marginal rotation [2]. Essential to managing the upper eyelid entropion is protecting the cornea by applying corneal trophic substances—eyewashes, gels and ointments [5]. The placement of a therapeutic contact lens is not a first intention solution because it is quickly expelled [1]. Another course of action that is not recommended is protecting the cornea with a dressing [5,7]. A therapeutic contact lens can be used with success in patient postoperative management.

Correcting a tarsal kink is eminently surgical—whether we are talking about open or closed procedures [2,3]. The goal is to weaken the tarsal fibrosis and to evert the eyelid margin. Multiple techniques have been described in case presentations. The surgery was a success every single time, indicating that the correction of the tarsal kink does not depend on the procedure [2]. The open procedures described are represented by: the excision of the fibrotic band associated with eversion sutures, the horizontal transection of the tarsal plate associated with eversion sutures, the repositioning of the anterior lamella, horizontal tarsotomy with marginal rotation, lamellar tarsoplasty and resection of the anterior lamella [1,3,4,5]. Closed procedures include different types of eyelid eversion sutures, intermarginal tarsorrhaphy and the mechanical extension of the tarsal plate followed by a temporary tarsorrhaphy on the grey line in the first few days of life [2,6].

The prognosis of the patients is very good in the case of early diagnosis and treatment, and irreversible corneal damage is thus avoided [1,2,6]. In this case, despite a relatively late intervention, after the installation of corneal damage, the result was very good, and we will continue to monitor the evolution of the patient’s visual acuity and refraction as they grow.

## 6. Conclusions

The congenital tarsal kink syndrome is a serious and rare disease that can have severe consequences for the patient if it is not treated properly and in time. The “invisibility” of the ciliary edge and the absence of the upper eyelid fold can be important diagnostic indicators for the neonatologist and pediatrician. A simple maneuver involving the eversion of the upper eyelid, revealing the posterior side of the eyelid, allows for early diagnosis. The tarsal kink must be taken into consideration when making the etiologic differential diagnosis of congenital corneal opacities and corneal ulcerations. Surgical treatment applied in time is effective and reduces the risk of corneal leucomas.

## Figures and Tables

**Table 1:** Characteristics of congenital tarsal kink syndrome reported cases.

Study	Patient Number	Age of Diagnosis	Gender	Laterality	Referral Diagnosis	Associations	Surgical Technique
Vahdani et al., 2016 [9]	1	9 days	Male	Right	Corneal opacity & red eye	Isolated	Everting sutures through a posterior approach
Naik et al., 2007 [2]	2	8 weeks	Male	Right	Congenital entropion	Isolated	Transconjunctival horizontal tarsotomy with marginal rotation
	3	4 weeks	Male	Left	Corneal scar	Isolated	Transconjunctival horizontal tarsotomy with marginal rotation
	4	1 week	Female	Right	Conjunctivitis	Isolated	Transconjunctival horizontal tarsotomy with marginal rotation
	5	6 weeks	Male	Left	Congenital entropion	Isolated	Transconjunctival horizontal tarsotomy with marginal rotation
	6	36 months	Male	Left	Corneal scar	Isolated	Transconjunctival horizontal tarsotomy with marginal rotation
	7	60 months	Male	Right	Corneal scar	Isolated	Transconjunctival horizontal tarsotomy with marginal rotation
Aziz et al., 2006 [1]	8	17 days	Female	Right	Congenital entropion	Distichiasis	Everting sutures
Batur et al., 2017 [4]	9	5 months	Male	Bilateral	Congenital entropion	Wiedemann–Rautenstrauch syndrome	Everting sutures
Lucci et al., 2003 [10]	10	2 months	Female	Right	Corneal opacity	Trisomy 13 (Patau syndrome) microcephaly, characteristic phenotype associated sloping forehead, flattened occiput, micrognathia, flat nose, short neck and atrial septal defect	Temporary eyelid margin suture
Demirel et al., 2012 [11]	11	3 months	Female	Right	Congenital entropion	Isolated	Modified temporary eyelid margin suture
McElevanney et al., 1994 [12]	12	7 days	Female	Bilateral	Corneal ulcer	Isolated	Unfold pressure
Price and Collins 1987 [3]	13	26 weeks	Male	Right	Congenital entropion	Isolated	Suture rotation
Sires 1999 [8]	14	Birth	Female	Left	Corneal ulcer	Isolated	Anterior lamellar reposition
	15	Birth	Female	Bilateral	Congenital entropion	Isolated	Kink resection, suture rotation
	16	Birth	Male	Right	Congenital entropion	Isolated	Suture rotation
	17	Birth	Male	Bilateral	Congenital entropion	Isolated	Suture rotation
	18	1 week	Female	Bilateral	Corneal ulcer	Isolated	Kink excision
	19	2 weeks	Male	Right	Congenital entropion	Isolated	Margin suture rotation
	20	2 weeks	Male	Bilateral	Corneal erosion	Cardiac defect	Levator repair, crease fixation
	21	4 weeks	Female	Right	Congenital entropion	Isolated	Incise kink, suture rotation, upper lid tightening
	22	4 weeks	Female	Left	Congenital entropion	Isolated	Incise kink, suture rotation
	23	4 weeks	Female	Left	Congenital entropion	Isolated	Incise kink, suture rotation
	24	6 weeks	Male	Left	Corneal ulcer	Isolated	Tarsal fracture and suture
	25	6 weeks	Male	Right	Corneal ulcer	Isolated	Posterior approach, kink excision
	26	10 weeks	Male	Right	Corneal ulcer	Isolated	Weaken kink, suture rotation, supratarsal rotation
	27	44 weeks	Male	Right	Corneal erosion	Isolated	Unfold kink, patch
